# Anti-p53 Autoantibody Detection in Automatic Glass Capillary Immunoassay Platform for Screening of Oral Cavity Squamous Cell Carcinoma

**DOI:** 10.3390/s20040971

**Published:** 2020-02-11

**Authors:** Yen-Heng Lin, Chih-Ching Wu, Wan-Ling Chen, Kai-Ping Chang

**Affiliations:** 1 Graduate Institute of Biomedical Engineering, Chang Gung University, Taoyuan 333, Taiwan; 2 Department of Otolaryngology-Head & Neck Surgery, Chang Gung Memorial Hospital, Linkou, Taoyuan 333, Taiwan; 3 Departments of Medical Biotechnology and Laboratory Science, Chang Gung University, Taoyuan 333, Taiwan; 4 Molecular Medicine Research Center, Chang Gung University, Taoyuan 333, Taiwan

**Keywords:** anti-p53 autoantibody, oral squamous cell carcinoma, enzyme-linked immunosorbent assay, microfluidics

## Abstract

The incidence of oral squamous cell carcinoma (OSCC), which is one of the most common cancers worldwide, has been increasing. Serum anti-p53 autoantibody is one of the most sensitive biomarkers for OSCC. Currently, the most commonly used method on clinical screening platforms is the enzyme-linked immunosorbent assay, owing to its high specificity and repeatability. However, conducting immunoassays on 96-well plates is typically time consuming, thereby limiting its clinical applications for fast diagnosis and immediate prognosis of rapidly progressive diseases. The present study performed immunoassays in glass capillaries of 1-mm internal diameter, which increases the surface to volume ratio of the reaction, to shorten the time needed for immunoassay. The immunoassay was automated while using linear motorized stages and a syringe pump. The results indicated that, when compared with the 96-well plate immunoassay, the glass capillary immunoassay decreased the reaction time from typical 120 min to 45 min, reduced the amount of reagent from typical 50 µL to 15 µL, and required only simple equipment setup. Moreover, the limit of detection for glass capillary anti-p53 autoantibody immunoassay was 0.46 ng mL^−1^, which is close to the 0.19 ng mL^−1^ value of the conventional 96-well plate assay, and the glass capillary method had a broader detection range. The apparatus was used to detect the serum anti-p53 autoantibody concentration in clinical patients and compare its results with the conventional 96-well plate method results, which suggested that both of the methods detect the same trend in the relative concentration of serum anti-p53 autoantibody in healthy individuals or patients with OSCC.

## 1. Introduction

Oral squamous cell carcinoma (OSCC) is the most common head and neck malignant neoplasm, whose incidence has been increasing recently [1]. In Taiwan, OSCC is the fifth most common cancer and the most common malignant neoplasm in males aged 30–50 years [2]. Although the past few decades have witnessed tremendous progress in the diagnosis and treatment of OSCC, approximately 40–50% of the patients died within five years of diagnosis [3], and the patient survival rate has not changed for 10 years, mainly because most patients have already been in the advanced stages of OSCC by the time of diagnosis, which has a poor prognosis and less than five years of survival time [4]. Therefore, early diagnosis can help to improve therapy and reduce the incidence of OSCC.

Several recent studies are searching for biomarkers that are suitable for early diagnostic screening. Some research has proposed autoantibodies as biomarkers for detecting cancers, such as colorectal cancer [5], breast cancer [6,7,8], ovarian cancer [9], lung cancer [10,11], and liver cancer [12,13], because of their high stability and specificity. Our team has investigated autoantibodies as biomarkers for the early detection of oral cavity squamous cell carcinoma and identified four most promising autoantibodies among several autoantibodies in saliva samples [14]. Among these four autoantibodies, anti-p53 autoantibody is the most sensitive one, which is detectable not only in saliva samples, but also in serum samples, as reported by previous studies [15,16,17]. In the present study, we chose anti-p53 as the screening biomarker and used human serum samples as the screening sample.

Several immunoassays have become available for the identification and quantification of biomarkers during the last few decades. These methods are dependent on the specific interaction between antibody and antigen to determine the concentration of the target substance in samples. The commonly used immunoassays include fluorescent immunoassay (FIA), chemiluminescence immunoassay (CLIA), and enzyme-linked immunosorbent assay (ELISA), with ELISA being the most widely used assay. ELISA is recognized as the gold standard in medical research for its high specificity and repeatability. However, the conventional 96-well plate ELISA has a few significant disadvantages, such as long detection time, complicated operating procedure, and demanding large quantities of reagents and samples [18,19], thereby limiting its clinical applications for fast diagnosis and immediate prognosis and resulting in missing the window of treatment, especially for rapidly progressive diseases, such as septicemia, acute organ rejection, and myocardial infarction.

Meanwhile, the development of microfluidic technology is making tremendous progress. For example, the protein chips made of polydimethylsiloxane (PDMS) integrate micropumps, micromixers, and microvalves to manipulate fluids for the automatic concentration determination of the target protein [20,21,22,23,24]. However, the design of such microfluidic chips usually involves complicated chip design and manufacture, and fluid control is a highly complex process. Moreover, failure in thorough washing during the assay often leads to problems of liquid residue and low repeatability. Some studies on microfluidic chips have used antibody-modified polystyrene beads or magnetic beads to purify the target protein and then detect the target protein by immunoreaction [25,26]. The tactic has the advantage of purifying samples before the assay, but the number of magnetic beads often decreases during the assay due to the washing and binding steps of the immunoreaction, thus reducing the accuracy and range of detectable concentration. In addition, some researches have harnessed microchannel disks, which control the fluid by centrifuge force, the geometry design of microchannels, and the surface hydrophilicity and hydrophobicity of the microchannels, to detect proteins [27,28,29]. However, the effective and homogenous modification of the surface of microchannels with antibodies to achieve stable detection remains challenging. For all of the methods described above, if the protein concentration determination involves optical signal detection, some bulky instruments such as a fluorescence microscope equipped with a photomultiplier tube reader or an ELISA reader are often necessary for detecting the result of reactions [30,31]. These types of setup diverge from the trend of miniaturization of detectors.

Based on the clinical demands for fast-detection microfluidic chips to have any clinical applications, the assay procedure and associated equipment must be simplified to achieve highly repeatable and stable sensitivity. In this study, immunoreactions were conducted in glass capillaries, only requiring simple fluid control. The use of linear motorized stages and a multi-channel syringe pump to handle specimen and reagents is sufficient for automatic immunoassay. The signal was detected with a cellphone camera instead of a fluorescence microscope or ELISA reader, thus further reducing the equipment requirement. The antigen–antibody reaction rate increases with less amount of sample and specimen because of the high surface to volume ratio of the capillary. Running the assay simultaneously in multiple capillaries might increase its throughput. Moreover, using autoantibodies as the target allows for a higher detection speed than that obtained with ELISA, because the method omits one round of antigen–antibody reaction and thus achieves a fast diagnosis. The anti-p53 autoantibody concentration in the specimen could be determined in 45 min by this method with 15 µL of sample. The end user does not need to be well trained in laboratory skills because the proposed platform can be developed as a compact and easy to use immunoassay platform like a point-of-care device. It is promising for the screening of oral cavity squamous cell carcinoma to facilitate early diagnosis.

## 2. Materials and Methods

### 2.1. Anti-p53 Antibody Detection and Experiment Set-Up

**Figure 1:**
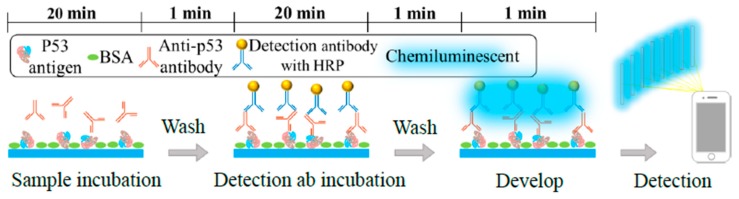
The experimental procedure and time table of the glass capillary immunoassay on autoantibodies. After the capillary was modified with the p53 antigen, the samples containing the anti-p53 antibody were transferred into the capillary, incubated for 20 min, and then washed three times to remove nonspecific antibody binding. Add the detection antibody into the capillary, incubate for 20 min, and then wash three times to remove the excess detection antibody; and finally, add the chemiluminescence substrate and record the result with a cellphone.

**Figure 2:**
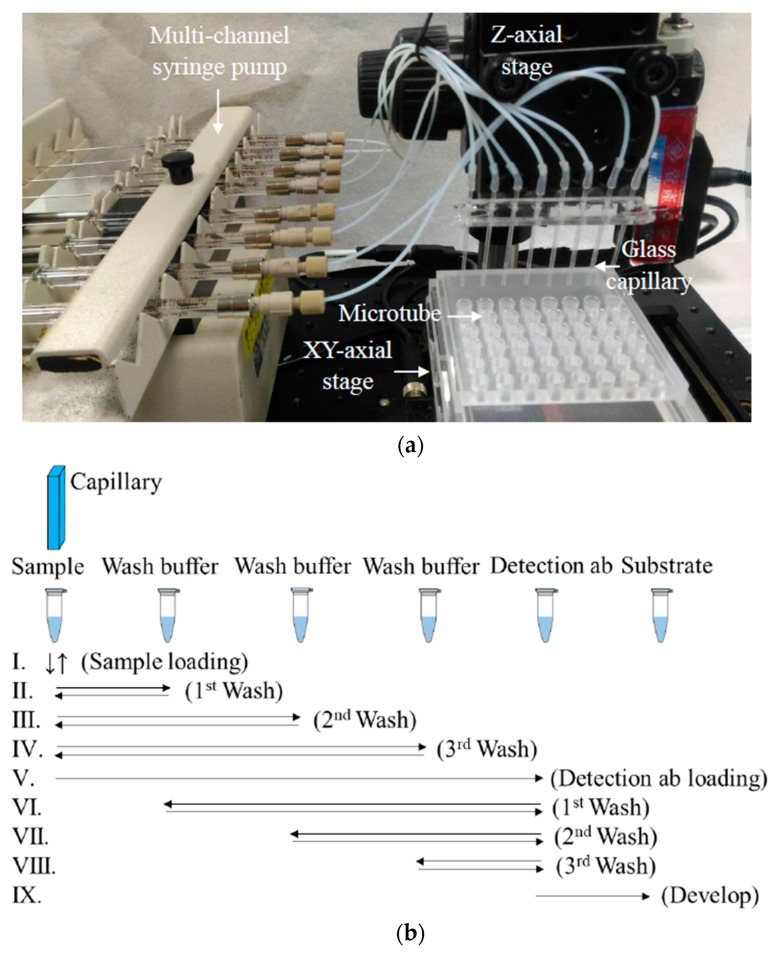
The experimental setting and control of the automatic glass capillary immunoassay platform. (**a**) The glass capillaries are held on the vertically movable Z stage and connected to a multi-channel syringe pump. Add the reagents and samples in the prearranged microcentrifuge tubes, which are carried by the motorized XY stage. The above setting is used to perform the immunoassay protocol outlined in (**b**) in the following order: sample loading, incubation, washing, detection antibody loading, incubation, washing, chemiluminescence substrate loading, and incubation.

Figure 1 shows the flowchart of the immunoreactions for detecting anti-p53 antibody in samples. The sample was sucked into a p53 antigen-modified glass capillary and then incubated for 20 min. A wash buffer removed the antibodies that did not bind to the antigen specifically. Subsequently, a HRP enzyme-linked detection antibody was introduced into the glass capillary and allowed to bond with the target antibody. After the wash buffer removed the excess detection antibody, a chemiluminescence substrate was added to complete the assay. The entire process was performed on an automatic motorized stage, as shown in Figure 2a. First, the p53 antigen-modified glass capillaries were fixed on the vertically movable Z stage (Zaber Technologies, Vancouver, Canada) and connected to a multi-channel syringe pump (KDS 230, KD Scientific, Holliston USA). The samples and reagents were preloaded into rows of 200 µL microcentrifuge tubes: the first row comprised samples at 20 µL/tube; the second, third, and fourth rows comprised the wash buffer at 200 µL/tube; the fifth row comprised the detection antibody at 20 µL/tube; and, the sixth row comprised the chemiluminescence substrate at 20 µL/tube. The XY stage (Zaber Technologies, Vancouver, Canada) moved the loaded microcentrifuge tubes during the procedure. Figure 2b shows the automatic assay process; the XY stage moved the sample tubes in the first row horizontally to align with the glass capillaries, and the Z stage and syringe pump then worked together to take the samples into the capillaries and incubate for 20 min before withdrawing the samples back to the original tubes. Next, the stage sequentially aligned the wash buffer tubes (in the second, third, and fourth rows) with the capillaries to transfer the wash buffer into the glass capillaries in the same manner as the previous step of sample taking. The used wash buffer was discarded into the microcentrifuge tubes in the first row. This washing step was repeated three times. Subsequently, the tubes in the fifth row were aligned with the glass capillaries and the detection antibody was sucked into the capillaries to provide a 20-min reaction time, followed by withdrawal of the antibody to their original tubes. The washing step was repeated three times between the second, third, fourth, and fifth rows of tubes. Finally, the chemiluminescence substrate tubes (the sixth row) were aligned with the glass capillaries, which then took up the substrate to react for 1 min and finished the automatic assay process.

### 2.2. Surface Modification of the Glass Capillaries

The hollow glass capillaries underwent surface modification to allow for antigen binding on the inner glass wall [32,33,34,35]. The glass capillaries were cleaned by a piranha solution (1:3 H_2_O_2_-concentrated H_2_SO_4_) before being coated by (3-aminopropyl) triethoxysilane (APTES). Subsequently, the dried glass capillaries were treated with oxygen plasma (30W, HARRICK PLASMA, Ithaca, USA) for 15 min to create hydroxyl groups (–OH) on the glass surface. The modification solution was prepared by dissolving APTES in acetone to reach a concentration of 30% (v/v). The glass capillaries were soaked overnight in the above solution with shaking to ensure a homogenous surface reaction. The capillaries were then washed with acetone to remove any unbound APTES and dried by heating at 100 °C for 30 min The APTES modified the amino groups (NH_2_) on the glass surface. The motorized stage setup that is described above was used to transfer 20 µL of antigen into the glass capillaries, which were then incubated for 3 h at 4 °C for a homogenous coating of p53 antigen. The amino groups on the modified glass surface have a strong affinity toward the carboxyl groups of antigens and can, therefore, bind the p53 antigen to the glass surface. The capillaries were washed four times with the wash buffer to remove excess p53 antigen and incubated in a blocking buffer for 20 min to block all of the unbound sites inside the capillary to prevent unspecific protein binding. Subsequently, the capillaries were washed four times and the capillaries were ready for anti-p53 autoantibody assay.

### 2.3. Reagents and Specimen

The hollow square glass capillaries used in the present study have an internal size of 1 × 1 mm with length of 10 mm and wall thickness of 0.2 mm (#8100-050, VitroCom, Mountain Lakes, USA). Acetone (J.T.Backer, USA) and APTES (440140, Sigma–Aldrich, USA) were used for capillary modification. The capillary coating antigen was recombinant human His_6_ p53 (SP-450, BostonBiochem, Cambridge, USA). The standard sample was prepared by dissolving mouse p53 antibody (SC-126, Santa Cruz Biotechnology, Dallas, USA) in phosphate-buffered saline (PBS, Sigma–Aldrich, USA). The wash buffer comprised PBS + 1% Tween 20, and the blocking buffer comprised PBS + 1% BSA. The chemiluminescence substrate was SupeSignal™ ELISA Femto Substrate (Thermo Fisher, USA). The mouse anti-p53 antibody and anti-mouse IgG-conjugated HRP (detection antibody) (NEF822001EA, PerkinElmer, USA) were used to setup the assay platform because pure human anti-p53 autoantibody was difficult to obtain. However, when detecting the anti-p53 autoantibody in the serum specimen from clinical patients, the anti-human IgG-conjugated HRP (PK-NE802001EA, PerkinElmer, USA) was used as the detection antibody. The present study tested four human serum specimens from four volunteers: two healthy controls (H1 and H2) and two patients with OSCC (P1 and P2). The patients with OCSS were diagnosed by histological analysis and physician routine examinations. The Institutional Review Board of Chi-Mei Medical Center approved this study (IRB number: 10012-L02), and the human serum specimens were collected with the consensus of the volunteers.

### 2.4. Capillary Chemiluminescence Detection and 96-Well Plate Immunoassay

The study used a chemiluminescene substrate in a glass capillary; therefore, the signal detection was conducted in a dark room to avoid interference from ambient light. The substrate interacts with the HRP enzyme-linked detection antibody to emit chemiluminescence at a stable intensity for 5 min before fading. The signal was recorded at 1 min after the reaction started while using a cellphone (iPhone 8 plus) with the following setting: f/1.8, exposure 1/4 s, ISO-2000, EV + 1.9, and focal length of 4 mm. The recorded images were quantitatively analyzed while using the Image J software. The chemiluminescent substrate has an emitting spectrum of approximately 420–520 nm. We only analyzed the blue channel to reduce the background noise. Moreover, only the average chemiluminescence intensity within 0.4 mm from the axis of the capillary was analyzed to avoid the inference of light reflection from the edge of the capillary.

The conventional 96-well plate assay was used with the same reagents, including the chemiluminescent substrate, to verify the performance of glass capillary assay. The differences were the incubation time and reagent volume. Note that the use of different substrates may cause different effects of signal yielding. Supplemental Materials Figure S1 shows the comparison of the chemiluminescent substrate and the TMS substrate for the same immunoassay. The white bottom plate (Sigma, Corning^®^ CLS3922) was used to avoid interference of unwanted light when conducting the plate assay with chemiluminescent substrate. The standard dose-response curves were built with the abovementioned method. In contrast, the commonly used gold standard plate assay with clear bottom plate (CLS9018, Corning) and NeA-Blue TMB substrate (Clinical Science Products Inc., USA) was used to compare the detection result in clinic sample to determine the possibility of detecting anti-53 autoantibody in clinical samples by the proposed glass capillary platform. The plate assay was performed, as follows: First, the 96-well plate surface was coated with the p53 antigen while shaking at 4 °C for overnight. On the next day, the plate was washed six times with the wash buffer, followed by blocking buffer incubation for 1.5 h and washing six times with the wash buffer. The standard sample or serum specimen was loaded in the wells and incubated for 1 h. The wells were washed six times before adding anti-mouse IgG-conjugated HRP or HRP-labeled anti-human IgG and then incubated for 40 min and washing three times. For obtaining the standard curve, chemiluminescent substrate was used and reacted with HRP in the dark for 1 min For the clinical sample test in the plate assay, the TMB substrate was added to react in the dark for 30 min The reaction was terminated with 2 N H_2_SO_4_ stop solution. An ELISA reader recorded the results of the plate assays performed while using two different substrates (SpectraMax M5 Microplate Reader, Molecular Devices, San Jose, USA). The parameters of plate assays were set as per our usual ELISA protocol. Note that, in our experience, low reagent volume would result in an unstable signal and a short incubation time might lead to relatively low sensitivity as compared with the usual protocol in plate assay.

## 3. Results and Discussion

### 3.1. Determination of p53 Antigen Concentration for Capillary Coating

**Figure 3:**
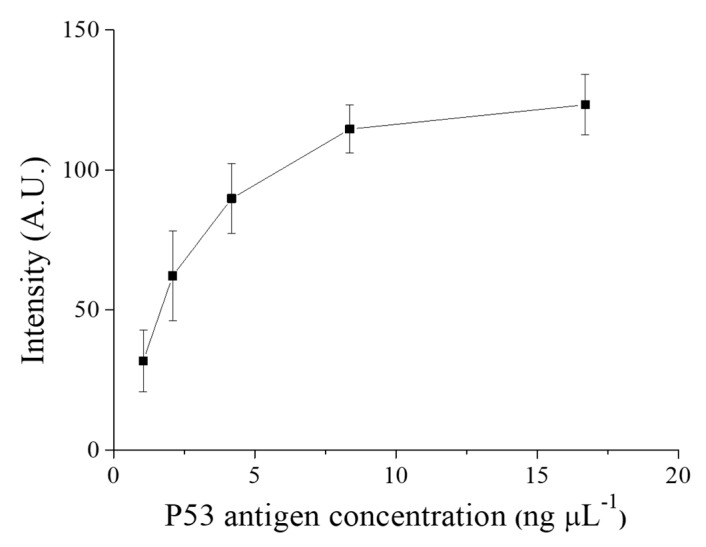
Optimization of the p53 antigen concentration for capillary glass modification. The glass capillaries were modified with p53 antigen at various concentrations of 16.7, 8.35, 4.18, 2.09, and 1.05 ng mL^−1^ and used for the immunoassay. The experiment was performed at fixed anti-p53 antibody and HRP-conjugated anti-mouse IgG concentrations with triplicates. The result indicates that the slope of the chemiluminescence intensity curve turns flat between p53 antigen concentrations of 8.36 and 16.7 ng µL^−1^.

First, we determined the optimal amount of P53 antigen for coating the capillary surface. Using excess of P53 antigen results in wastage, whereas using little antigen reduces the detection efficiency. The idea was to apply a slightly excess amount of p53 antigen to saturate the capillary glass surface. Based on the 96-well plate coating protocol that was provided by the manufacturer, the surface area of each well, and the surface area of a glass capillary that can be covered by 20 µL of antigen. We deduced a theoretical value of antigen concentration needed for coating the glass capillary as 16.7 ng mL^−1^; starting from this concentration, a serial dilution was obtained for antigen concentrations of 8.35, 4.18, 2.09, and 1.05 ng mL^−1^. These five antigen concentrations were used to coat the glass capillaries by the method that was described in the Section 2.2. The coated capillaries were tested in the immunoassay with 400 ng mL^−1^ p53 antibody and followed by the addition of 500 ng mL^−1^ detection antibody (HRP-conjugated anti-mouse IgG). Each incubation time for the immune reactions was 20 min Finally, the chemiluminescence substrate was allowed to react with the immune complex for 1 min in the capillaries. Glass capillary without the coating of p53 antigen was used as negative controls. Each antigen concentration was tested with three replicates, and the chemiluminescence data were plotted after subtracting the respective negative control. The signal intensity of negative control was quite low, which was 1.12 ± 1.14 A.U. (STD, n = 3). The chemiluminescence intensity increased with the increase in p53 antigen coating concentration until the antigen concentration reached 8.36 ng mL^−1^, at which the chemiluminescence intensity curve turned flat, indicating that p53 antigen had occupied all the available binding sites on the surface of the glass capillary, as shown in Figure 3. We used 16.7 ng mL^−1^ as the p53 antigen coating concentration in the subsequent experiments to ensure a saturated p53 antigen coating.

### 3.2. Immunoassay Incubation Time Optimization in Capillary

**Figure 4:**
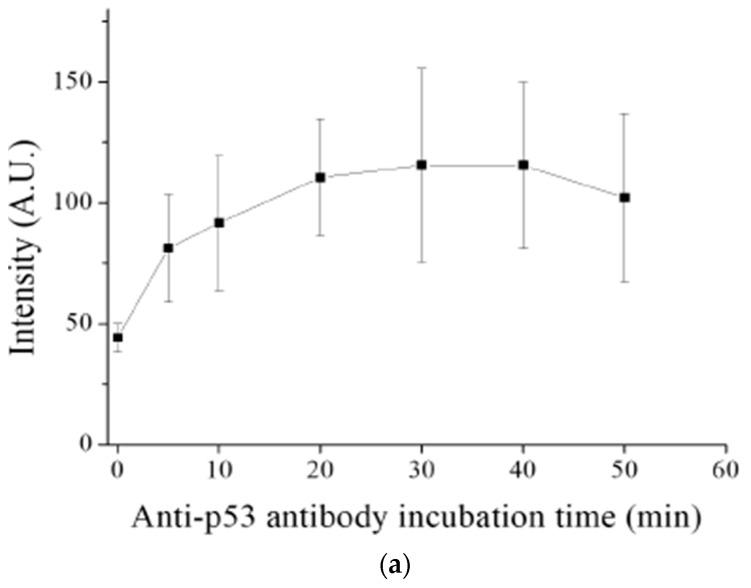

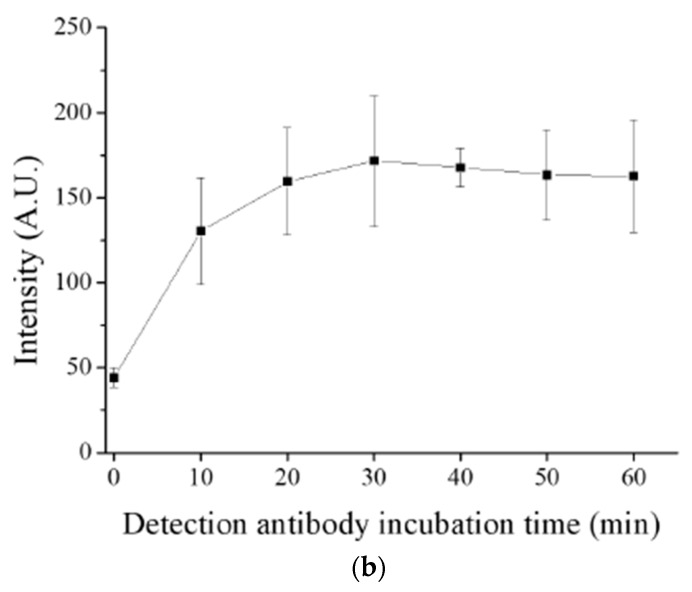
Optimization of incubation time for antigen–antibody interaction. (**a**) Change the incubation time for p53 antigen and anti-p53 antibody interaction, (**b**) change the incubation time for anti-p53 antibody and HRP-conjugated anti-mouse IgG interaction. All immunoassays were carried out with triplicates at fixed anti-p53 antibody and HRP-conjugated anti-mouse IgG concentrations.

The glass capillary assay increases the chances of interaction between the reagents owing to the shorter distance of diffusion and higher surface area to volume ratio, thus reducing the reaction time of the immunoassay when compared with the 96-well plate. We conducted experiments with the following settings to determine the optimal incubation times for the reaction between the antigen (on the capillary surface) and the anti-p53 antibody (in the specimen) and the reaction between the anti-p53 antibody and the HRP-conjugated anti-mouse IgG: the concentrations of the anti-p53 antibody and the HRP-conjugated anti-mouse IgG were 400 and 500 ng mL^−1^, respectively. The HRP-conjugated anti-mouse IgG incubation time was fixed at 20 min, whereas the incubation time for the reaction between the antigen and anti-p53 antibody varied at 5, 10, 20, 30, 40, and 50 min when testing the incubation time for the reaction between the antigen on the glass capillary and the anti-p53 antibody in the specimen. Each time setting was tested while using three replicates. As shown in Figure 4a, the chemiluminescence intensity increased along with the anti-p53 antibody incubation time, especially during the initial 20 min The extent of increase reduced after 30 min, and the chemiluminescence intensity decreased slightly after 40 min The increase in the chemiluminescence over time was possibly caused by the amount of antigen on the capillary wall that was occupied by the antibody during that incubation period. The reaction reached dynamic equilibrium in approximately 20–30 min. We also tested the incubation time for the reaction of the anti-p53 antibody with the HRP-conjugated anti-mouse IgG since the affinity between antibodies might be different. We fixed the antigen to anti-p53 antibody incubation time at 20 min, whereas the incubation time for the reaction between the anti-p53 antibody and the HRP-conjugated anti-mouse IgG varied at 10, 20, 30, 40, 50, and 60 min. The saturation time for the reaction between the anti-p53 antibody and the HRP-conjugated anti-mouse IgG was similar to that for the reaction between the p53 antigen and the anti-p53 antibody described above, suggesting that the molecular affinity involved in these two reactions are comparable, as shown in Figure 4b. Therefore, the incubation time was set at 20 min for both reactions in this study. Note that the detection signal of the 0 min incubation was achieved by sucking the sample into the coated capillary and then immediately flushing it out. The signal levels of 0 min incubation in both anti-p53 antibody and detection antibody testing were approximately 50 units, which were used to confirm that the signal was produced by immunoreaction and not by the background noise. The capillary has a smaller internal size and a larger specific surface area than the 96-well plate, thus reducing the incubation time for the antigen–antibody reaction from 60 min to 20 min and shortening the overall time for the assay.

### 3.3. Comparison between the Glass Capillary and 96-Well Plate Methods in Anti-p53 Antibody Detection

**Figure 5:**
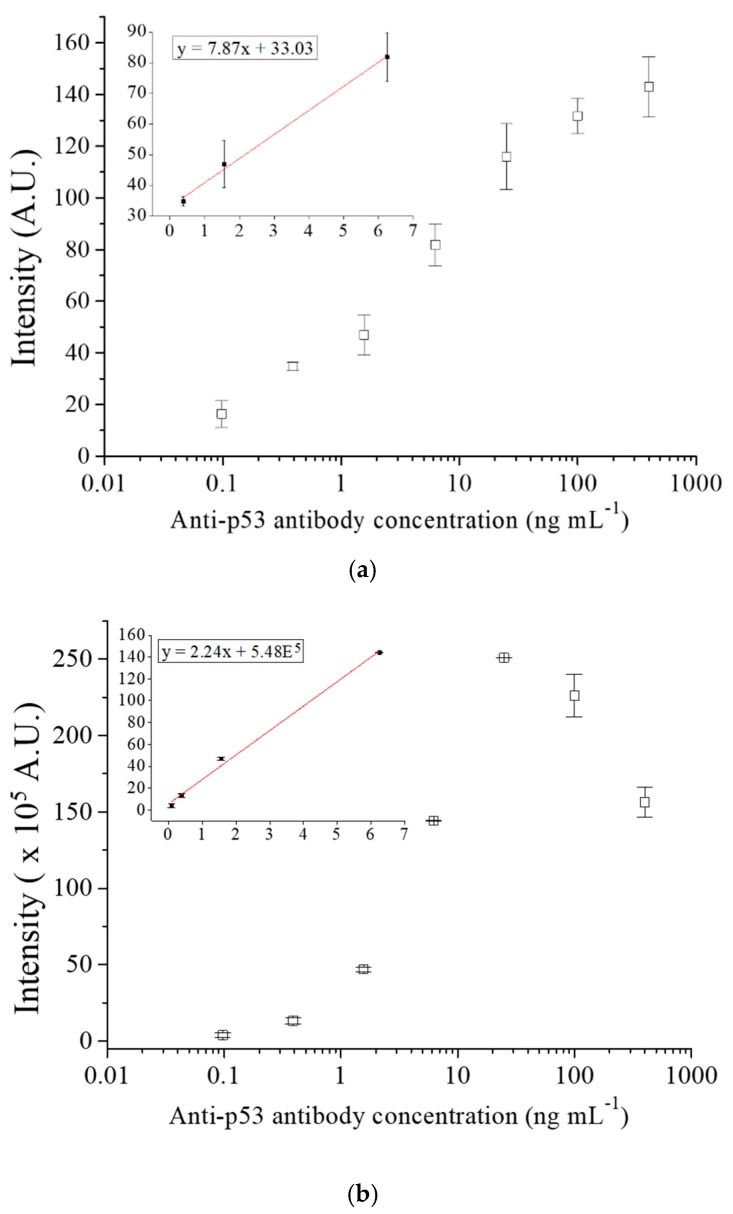
(**a**) The anti-p53 antibody standard curve assay in the glass capillary. The anti-p53 antibody standard solutions at various concentrations, including 400, 100, 25, 6.25, 1.56, 0.39, and 0.098 ng mL^−1^, were tested. (**b**) The anti-p53 antibody standard curve assay in the 96-well plate as a comparison to the above glass capillary method.

We compared the performance of anti-p53 antibody detection between the glass capillary and 96-well plate assay to validate the efficiency of the glass capillary assay. We conducted the same immunoassay process along with the reagent on both the platforms with different incubation times and amounts of reagents. In the preliminary study, the signal reached a plateau when the concentration exceeded 400 ng m mL^−1^, as shown in Supplemental Materials Figure S2. Therefore, a 400 ng mL^−1^ anti-p53 antibody standard specimen was prepared, and a set of 1:4 serial dilutions were made to obtain seven concentrations (400, 100, 25, 6.25, 1.56, 0.39, and 0.098 ng mL^−1^). Each concentration was tested with three replicates. The negative controls were set at 0 ng mL^−1^ anti-p53 antibody in PBS as a sample. The average intensity of detection signal was calculated by the detected signal subtracting the respective negative control. The signal of negative control of the capillary immunoassay was 3.03 ± 1.12 A.U. (STD, n = 3). Figure 5a shows the results of the antibody assay in glass capillaries and Figure 5b shows the results of the antibody assay in 96-well plates. The inset of Figure 5a expresses the linear part of the standard curve (from 0.39 to 6.25 ng mL^−1^) of the glass capillary assay, whereas the inset of Figure 5b expresses the same part (from 0.098 to 6.25 ng mL^−1^) of the plate assay. The graphs indicate that both of the platforms had similar trends of reaction and that the chemiluminescence intensity increased with the antibody concentration. The glass capillary assay appears to have a wider detection range than the 96-well plate assay. The detection range of the glass capillary assay was 0.46–400 ng mL^−1^, whereas that of the 96-well plate assay was 0.19–25 ng mL^−1^, probably because the capillary has a higher specific surface area. The LOD was calculated while using the formula: LOD = 3 × SD/S [36,37], where SD is the standard deviation of the three tests at the specimen concentration of zero and S is the slope of the linear part of the standard curves. The capillary platform LOD was approximately 0.46 ng mL^−1^ and the 96-well plate LOD was approximately 0.19 ng mL^−1^. The capillary platform LOD was higher because of a high standard deviation among the experimental replicates, which might be because of the uneven coating of p53 antigen on the surface of the capillaries. As for the detection time and required sample volumes, the total assay time decreases because the glass capillary has a higher specific surface area and a shorter distance of diffusion to increase the rate of reaction. Moreover, the small volume of the capillary reduces reagent use. It took approximately 45 min from loading the sample to reading the results on the capillary platform, which was faster than the 120 min that was required for the typical 96-well plate assay. The capillary also used a smaller amount of sample (15 µL) than the 96-well plate (50 µL). Table 1 compares the results between the two platforms. Furthermore, the detection range is broadly comparable and the LOD is higher than the electrochemical platform for approximately one order when compared to the detection by an electrochemical impedimetric sensor with gold electrode for anti-p53 autoantibody [38]. A research group demonstrated the use of an Au nanoparticle-decorated graphene nanosheet as a electrochemical immunosensor for the detection of anti-p53 autoantibody [39]. The LOD in such platform is relatively low, with 0.1 pg mL^−1^. This might be due to the fact that the graphene-based/Au nanocomposites electrode has high electrical conductivity and enhanced surface area. However, the electrochemical sensor needs a relatively expansive instrument, such as potentiostat/galvanostat, to read a tiny electrical current. This might not be easily available in common clinical laboratories or hospitals.

### 3.4. Patient Serum Sample Test

**Figure 6:**
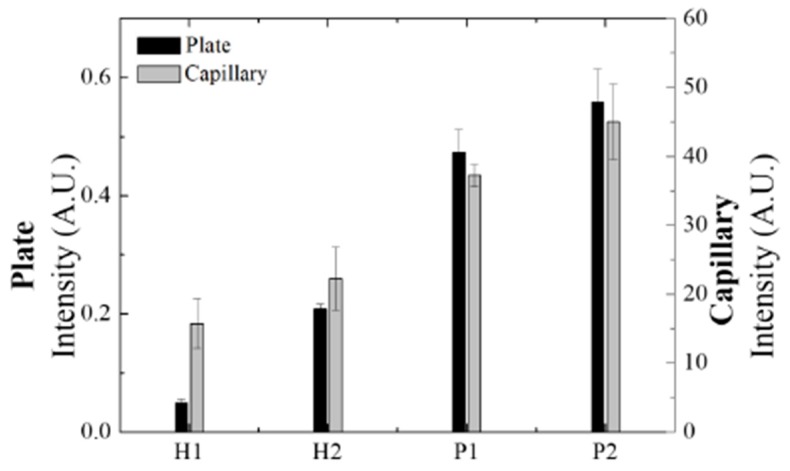
The feasibility evaluation of using glass capillaries for clinical serum specimen anti-p53 autoantibody immunoassay.

We compared the capillary platform and the 96-well plate on basis of their performance of detecting serum anti-p53 autoantibody while using the serum specimens from volunteers as samples to evaluate the feasibility of using the glass capillary immunoassay in clinical specimen examination. The serum specimens from four volunteers were tested on both the platforms, including two healthy controls (H1 and H2) and two patients with OSCC (P1 and P2). The experimental setting is the same as described in the Section 3.3, except the use of TMB as the substrate in plate assay. The serum specimens were diluted 2000 times and tested with three replicates. The gray columns represent the results from the capillary platform with the right Y-axis indicating the chemiluminescence intensity, as shown in Figure 6; the black columns represent the results from the conventional 96-well plate with the left Y-axis indicating the optical density. The molecular affinity involved might be different from that between the mouse anti-p53 antibody and the anti-mouse IgG-conjugated HRP used to build the assay platform because the detection antibody for the patient serum specimens was HRP-labeled anti-human IgG. Theoretically, the affinity between commercially available mouse anti-p53 antibody and anti-mouse IgG would be stronger than anti-p53 autoantibody and anti-human IgG. The calibration curve set by mouse source antibodies cannot be used to calculate the relative concentration of anti-p53 autoantibody in serum that was detected by anti-human IgG. Therefore, the results of the serum samples from patients with OSCC are shown as relative concentrations. The results of previous studies have shown that most OSCC patients have significantly higher anti-p53 autoantibody concentrations than in healthy controls. Others may have small differences. In addition, healthy volunteers also have different concentrations of anti-p53 antibodies in the serum [14,15,16,17]. Both of the assays demonstrated that P1 and P2 emitted stronger signals than H1 and H2, suggesting that it is feasible to use the glass capillary platform for serum samples, as shown in the Figure 6. The large difference for H1 in the two assays may due to the low concentration of anti-p53 autoantibody in the sample. The lower concentration leads to a larger variation in the detection signal in both assay platforms.

The specimens from four volunteers were tested, including two healthy controls (H1 and H2) and two patients with OSCC (P1 and P2). Each specimen was screened by both platforms with triplicates. The results show that, on both platforms, the signals from the serum specimens P1 and P2 were stronger than that from the serum specimens H1 and H2.

## 4. Conclusions

In this study, we developed an automatic immunoassay platform that uses hollow square glass capillaries to detect the anti-p53 autoantibody as a biomarker of OSCC in the serum specimens. The capillary platform performed the assay in approximately 45 min, while the reducing reagent used was 15 µL. The entire assay platform is under the automatic control of an XYZ motorized stage and a syringe pump to reduce human operation errors. The chemiluminescence assay results were recorded using a cellphone, which is a simplified modification in the result reading method as compared with the ELISA reader or camera. The glass capillary assay platform was further tested with the clinical serum specimens from patients with OSCC and its results were compared with the conventional 96-well method results, which showed that glass capillary assay platform can detect the relative content of anti-p53 autoantibody in serum samples from healthy individuals and patient with OSCC, thus proving the clinical feasibility of the platform. At present, the major issue with the glass capillary immunoassay platform is the high variation, which was occasionally observed in the triplicate experiments. The possible reason is the lack of control to ensure a homogenous coating of p53 antigen on the glass surface as well as the modification of the glass surface while using APTES. The covalent cross-linking between the p53 antigen and capillary surface might be a method for reducing variation. The density of hydroxyl groups on the surface and the concentration of APTES can also influence the density of binding sites quantity of molecules deposed. The stability of the proposed detection platform might be further improved by addressing such issues.

## Figures and Tables

**Table 1:** Comparison of LOD, detection range, assay time, and sample volume between the anti-p53 autoantibody assays of glass capillary platform and typical protocol of 96-well plate.

	LOD (ng mL^−1^)	Detection Range (ng mL^−1^)	Assay Time (min)	Sample Volume (µL)
Glass capillary immunoassay	0.46	0.46–400	45	15
96-well plate assay	0.19	0.19–25	120	50

LOD: limit of detection.

## Supplementary Materials

The following are available online at https://www.mdpi.com/1424-8220/20/4/971/s1, Figure S1: The effects of TMB and chemiluminescent substrates in plate assay, Figure S2: The detection signal of concentrations of anti-p53 antibody from 40,000 to 0.4 ng/mL.
