# Supplementary material for: Anti-p53 Autoantibody Detection in Automatic Glass Capillary Immunoassay Platform for Screening of Oral Cavity Squamous Cell Carcinoma
Source: Sensors (Basel). 2020 Feb 11;20(4):971. doi: 10.3390/s20040971 (PMC7070657; doi:10.3390/s20040971)
Supplement: Supplementary file 1 [file sensors-20-00971-s001.pdf]

sensors

Article

Anti-p53 Autoantibody Detection in Automatic Glass Capillary Immunoassay Platform for Screening of Oral Cavity Squamous Cell Carcinoma

Yen-Heng Lin [1,2,*,†], Chih-Ching Wu [2,3,4,†], Wan-Ling Chen [1] and Kai-Ping Chang [2,4]

1 Graduate Institute of Biomedical Engineering, Chang Gung University, Taoyuan 333, Taiwan
2 Department of Otolaryngology-Head & Neck Surgery, Chang Gung Memorial Hospital, Linkou, Taoyuan 333, Taiwan
3 Departments of Medical Biotechnology and Laboratory Science, Chang Gung University, Taoyuan 333, Taiwan
4 Molecular Medicine Research Center, Chang Gung University, Taoyuan 333, Taiwan
* Correspondence: yenheng@mail.cgu.edu.tw; Tel.: +886-3-2118800 (ext. 3789); Fax: +886-3-2118507
† These authors contributed equally to this work.

Received: 3 December 2019; Accepted: 8 February 2020; Published: 11 February 2020

The effects of TMB and chemiluminescent substrates in plate assay for the anti-p53 antibody detection. A set of 10 times serial dilutions anti-p53 antibody standard sample was made to obtain six concentrations (400, 40, 4, 0.4, 0.04, and 0.004 ng mL^{-1}) for testing. Both the plate assay procedures were the same except the substrates reaction. As shown in Figure S1, the TMB substrate has higher sensitivity than chemiluminescent substrate in the concentration range of 0.004 to 4 ng mL^{-1}. This may be due to the longer reacting time of the TMB substrate.

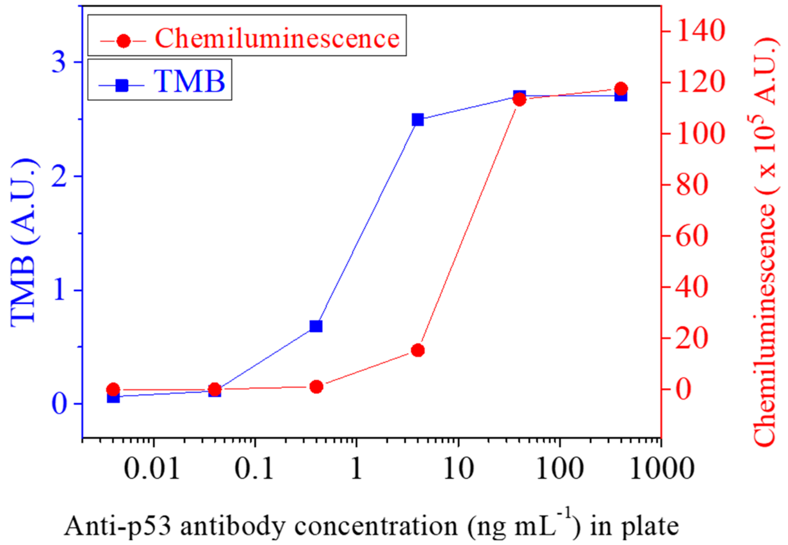

Figure S1. The effects of TMB and chemiluminescent substrates in plate assay.

The concentrations of anti-p53 antibody were tested from 40000 to 0.4 ng/mL with 10 times series dilution. Figure S2 shows the quantitative analysis of different p53 antibody concentrations after the assay was developed with chemiluminescence substrate in glass capillary. The chemiluminescent intensity reached to a plateau when the concentration exceeded 400 ng/mL.

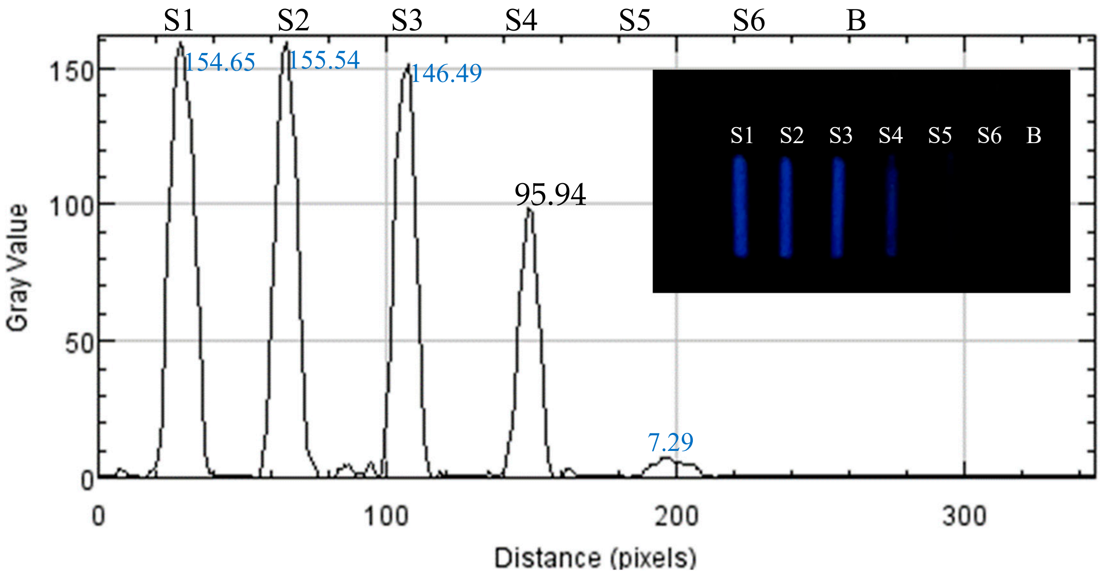

Figure S2. The detection signal of concentrations of anti-p53 antibody from 40000 to 0.4 ng/mL.

P53 antibody concentration; S1: 40000 ng/mL; S2: 4000 ng/mL; S3: 400 ng/mL; S4: 40 ng/mL; S5: 4 ng/mL; S6: 0.4 ng/mL; Blank: 0 ng/mL.
